# Cardiac arrest and post-discharge mortality in patients with myocardial infarction: A large-scale nationwide registry analysis

**DOI:** 10.1016/j.resplu.2024.100647

**Published:** 2024-05-03

**Authors:** Hirohiko Ando, Mitsuaki Sawano, Shun Kohsaka, Hideki Ishii, Atomu Tajima, Wataru Suzuki, Ayako Kunimura, Yusuke Nakano, Ken Kozuma, Tetsuya Amano

**Affiliations:** a Department of Cardiology, Aichi Medical University, Nagakute, Japan; b Section of Cardiovascular Medicine, Department of Internal Medicine, Yale School of Medicine, Yale New Haven Hospital Center of Outcomes Research and Evaluation, New Haven, CT, USA; c Department of Cardiology, Keio University School of Medicine, Tokyo, Japan; d Department of Cardiovascular Medicine, Gunma University Graduate School of Medicine, Maebashi, Japan; e Department of Cardiology, Teikyo University, Tokyo, Japan

**Keywords:** Acute myocardial infarction, Cardiac arrest, Post-discharge mortality, Prognosis, J-PCI OUTCOME registry

## Abstract

**Background:**

Cardiac arrest is a serious complication of acute myocardial infarction. The implementation of contemporary approaches to acute myocardial infarction management, including urgent revascularization procedures, has led to significant improvements in short-term outcomes. However, the extent of post-discharge mortality in patients experiencing cardiac arrest during acute myocardial infarction remains uncertain. This study aimed to determine the post-discharge outcomes of patients with cardiac arrest.

**Methods:**

We analysed data from the J-PCI OUTCOME registry, a Japanese prospectively planed, observational, multicentre, national registry of percutaneous coronary intervention involving consecutive patients from 172 institutions who underwent percutaneous coronary intervention and were discharged. Patients who underwent percutaneous coronary intervention for acute myocardial infarction between January 2017 and December 2018 and survived for 30 days were included. Mortality in patients with and without cardiac arrest from 30 days to 1 year after percutaneous coronary intervention for acute myocardial infarction was compared.

**Results:**

Of the 26,909 patients who survived for 30 days after percutaneous coronary intervention for acute myocardial infarction, 1,567 (5.8%) had cardiac arrest at the onset of acute myocardial infarction. Patients with cardiac arrest were younger and more likely to be males than patients without cardiac arrest. The 1-year all-cause mortality was significantly higher in patients with cardiac arrest than in those without (11.9% vs. 2.8%, *p* < 0.001) for all age groups. Multivariable analysis showed that cardiac arrest was an independent predictor of all-cause long-term mortality (hazard ratio: 2.94; 95% confidence interval: 2.29–3.76).

**Conclusions:**

Patients with acute myocardial infarction and concomitant cardiac arrest have a worse prognosis for up to 1 year after percutaneous coronary intervention than patients without cardiac arrest.

## Highlights

• Among patients who survive 30 days after AMI, all-cause mortality at 1 year is higher in patients with CA than in those without CA.
• Continued monitoring and targeted intervention are needed for these high-risk patients.
• Further research is needed to determine the best treatment strategies for patients with CA during the vulnerable period.

## Introduction

The management of acute myocardial infarction (AMI) has significantly improved in recent decades largely owing to the increased application of revascularization procedures such as primary percutaneous coronary intervention (PCI).^1^, ^2^ However, despite these advances, certain high-risk subgroups of patients with AMI have a poor prognosis. Cardiac arrest (CA) is the most devastating manifestation of AMI. CA occurs in approximately 6–9% of patients treated with primary PCI, with younger patients having a higher risk of CA than older patients.^3^, ^4^, ^5^, ^6^ Recent efforts have focused on improving *peri*-resuscitation care, including raising awareness on prehospital cardiopulmonary resuscitation, improvements in mechanical circulatory support devices, and the use of targeted temperature management.^7^, ^8^, ^9^, ^10^, ^11^, ^12^ Consequently, a trend toward a gradual improvement in short-term mortality after out-of-hospital CA has been reported.^13^, ^14^, ^15^

In addition to assessing the short-term prognosis, evaluating the long-term prognosis of patients with CA at the onset of AMI is crucial. The availability of prognostic information facilitates the decision-making process including the development of a comprehensive monitoring plan and tailored rehabilitation strategy. It also provides patients and their families with accurate and more granular information about potential future adverse events. Specifically, identifying a “vulnerable” period for patients with CA at the onset of AMI following hospital discharge would provide valuable insights and enhance our understanding of long-term outcomes.

To date, there have been several reports on the long-term prognosis of patients with CA after discharge. These suggest that post-discharge mortality is comparable between patients with and without CA.^16^, ^17^ However, these reports included a relatively small number of patients and used outdated registries and do not reflect current *peri*-resuscitation practice. Evaluations of post-discharge prognosis in large numbers of patients with CA treated using contemporary management are needed. The present study aimed to determine the post-discharge prognosis of patients with CA at the onset of AMI using the most contemporary nationwide large-scale registry database.

## Methods

### Ethical approval

The study protocol was approved by an independent central ethics committee at the Clinical Research Promotion Network Japan and by local institutional review boards at each site. All procedures were conducted in accordance with the tenets of the Declaration of Helsinki. Informed consent was obtained from the patients through an opt-out format notice on websites and posters.

### Study design and data source

The present analysis utilized data from the J-PCI OUTCOME, an ongoing, prospectively planned observational multicentre, nationwide PCI registry led by the Japanese Society of Cardiovascular Intervention and Therapeutics (CVIT). Details of the J-PCI OUTCOME have been previously published.^18^, ^19^ The J-PCI OUTCOME was established to evaluate the 1-year results of the original J-PCI Registry. This registry was designed to provide national, regional, and institutional information on general patient characteristics, including the background, clinical presentation, angiographic and procedural details, and in-hospital death within 30 days of PCI.^6^, ^20^, ^21^ Cardiac catheterization procedures are performed in both public and privately funded hospitals in Japan. Since both systems require registration in the J-PCI Registry for certification application and renewal, more than 80% of all PCIs performed in Japan are registered in the J-PCI Registry.^20^ Today, >200,000 PCI cases are registered annually by approximately 900 institutions, representing >90% of PCI-performing hospitals in Japan.^22^, ^23^ Each hospital has a data manager who is responsible for collecting and entering PCI data into the online database.

The J-PCI OUTCOME includes patients from the J-PCI Registry who are discharged alive. J-PCI OUTCOME invited institutions that met the eligibility criteria of a minimum of 200 PCI cases per year at each site to participate in the study. Of these, 179 hospitals that volunteered to participate proceeded with the research protocol. Between 2017 and 2018, the period analyzed in this study, 105,592 patients were enrolled in J-PCI OUTCOME, representing 20.4% of all patients enrolled in the J-PCI registry (*n* = 517,735) during the same period. Patients who died within 30 days of the index PCI were excluded from the analysis of 1-year patient outcomes. Predefined prognostic events include all-cause and cardiac death, as well as data on other adverse events, including non-fatal events requiring hospitalization for acute coronary syndrome (ACS), stroke, bleeding, acute heart failure and repeat revascularization. Prognostic data were collected by either a review of medical records or through telephone interviews by data entry personnel and then reviewed by the data managers at each site. These collected data were extracted and analysed for the present study.

### Variable definitions

AMI was defined as persistent symptoms of myocardial ischemia accompanied by elevated levels of cardiac markers according to the J-PCI protocol.^23^ Cardiac biomarkers included creatine kinase or creatine kinase-MB and troponin, with elevations defined as a two-fold increase over normal values and levels ≥99th percentile, respectively. CA was defined as asystole, ventricular fibrillation, or pulseless ventricular tachycardia requiring cardiopulmonary resuscitation within 24 h before PCI. This included both out-of-hospital and in-hospital cardiac arrest that occurred before PCI. Acute heart failure was defined as symptoms of heart failure within 24 h before PCI. These symptoms included dyspnoea on mild activity, orthopnoea, body fluid retention, wet rales, jugular venous distention, and pulmonary oedema. Hypertension, diabetes, dyslipidaemia, and chronic kidney disease (CKD) were defined as described previously.^23^

### Clinical outcomes

Clinical endpoints were defined according to the 2017 Cardiovascular and Stroke Endpoint Definitions for Clinical Trials.^24^ Definite stent thrombosis was adjudicated for cardiovascular death and non-fatal ACS events according to the Academic Research Consortium-2 Consensus Document.^25^ The causes of death were categorized as non-cardiac death; death from an unknown cause; cardiac death with subcategories of death due to ACS, sudden cardiac death, and death due to heart failure; death due to stroke; death due to a procedural complication; and death due to bleeding events. Patients with ACS were subdivided into those presenting with ST-elevation myocardial infarction (STEMI), non-STEMI, and unstable angina with or without revascularization. Bleeding events were subdivided into access site bleeding, upper gastrointestinal bleeding, lower gastrointestinal bleeding, cerebrovascular or spinal bleeding, genitourinary bleeding, and bleeding of an unknown origin.

### Statistical analysis

Baseline patient and coronary lesion characteristics, details of specific procedures used, and 1-year outcomes were summarized as the mean ± standard deviation, median and interquartile range, or counts and percentages, as appropriate. Differences between the patients with and without CA at the onset of AMI were analysed using Student’s *t*-test or the Wilcoxon rank sum test for continuous variables and the Chi-square test for categorical variables, as appropriate. Kaplan–Meier curves were generated to estimate all-cause mortality from 30 days to 1 year after PCI for the overall cohort and for age-based subgroups (<60 years, 61–70 years, and 71–80 years), and the log-rank test was used to assess whether the differences between the outcome curves were significant (i.e., *p* < 0.05).

To determine the relationship between CA and all-cause death, multivariable Cox proportional hazards modelling was performed. Covariables used in this model were selected based on clinical judgment and a literature review and included the following: CA, age, male sex, STEMI, left main trunk lesion, multivessel disease, hypertension, diabetes, dyslipidaemia, current smoking status, and CKD. The hazard ratios for each variable are reported along with the 95% confidence intervals (CIs). Patients with missing data were excluded from the multivariable analyses. All data were managed by the National Clinical Database and by data analysts from the J-PCI Registry Statistical Analysis Team. All analyses were performed using Stata/IC Version 15.1 for Macintosh (StataCorp, College Station, TX, USA). All *p* values were two-tailed, and *p* < 0.05 indicated statistical significance.

## Results

### Patient demographics and lesion characteristics

**Fig. 1:**
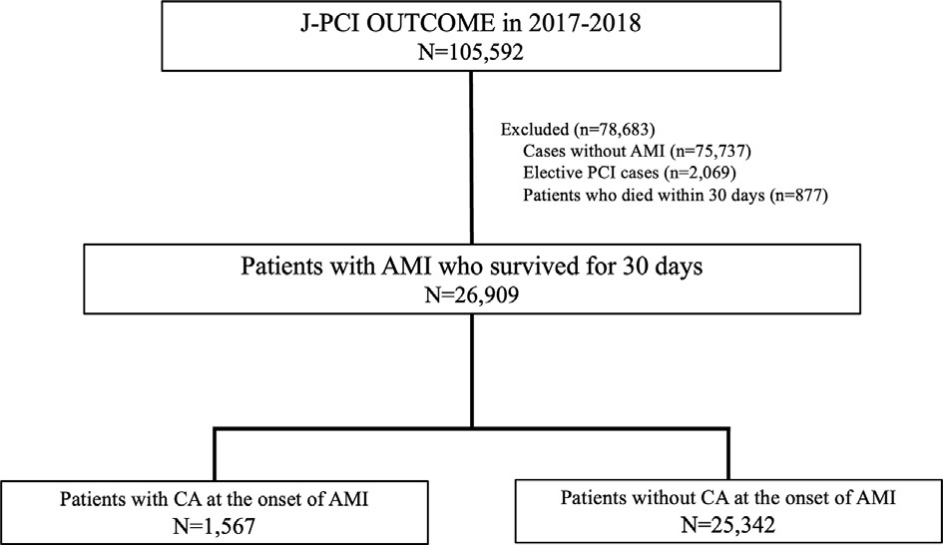
**Study flow chart.** This study included J-PCI OUTCOME data from 2017 and 2018 (*N* = 105,592). Of the 26,909 patients surviving for 30 days after primary PCI, 1,567 (5.8%) experienced CA at presentation and 25,342 (94.2%) did not. AMI, acute myocardial infarction, CA, cardiac arrest, PCI, percutaneous coronary intervention.

Of the 105,592 patients enrolled in J-PCI OUTCOME in 2017 and 2018, 26,909 were finally included in the study's analysis after excluding cases without AMI, elective PCI cases, and patients who died within 30 days. 1,567 (5.8%) experienced CA at the onset of AMI and 25,342 (94.2%) did not (Fig. 1). The baseline demographics, lesion characteristics, and procedural details are summarized in Table 1. Patients with CA were younger and more likely to be males and had a lower prevalence of traditional coronary risk factors (e.g., hypertension and diabetes) but a higher prevalence of CKD and dialysis than did those without CA. With respect to myocardial infarction, the proportion of STEMI was significantly higher in patients with CA than in those without, although the difference was not significant. The prevalence of cardiogenic shock and transfemoral access was significantly higher and the use of mechanical assist devices was more frequent in patients with CA, reflecting the clinical manifestations of CA. Angiographic findings showed that patients with CA had more complex lesions such as multivessel disease and left main trunk lesions.

**Table 1:** Baseline demographics, lesion characteristics, and procedure details.

Characteristics	Total *N* = 26,909	Patients with CA *N* = 1,567	Patients without CA *N* = 25,342	*p* value
Age, years	69 (13)	67 (13)	69 (13)	<0.001
Age group				<0.001
≤60 years	6,930 (26%)	475 (30%)	6,455 (25%)	
61–70 years	7,304 (27%)	499 (32%)	6,805 (27%)	
71–80 years	7,313 (27%)	362 (23%)	6,951 (27%)	
≥81 years	5,362 (20%)	231 (15%)	5,131 (20%)	
Male sex	20,685 (77%)	1,296 (83%)	19,389 (77%)	<0.001
Hypertension	18,247 (68%)	932 (59%)	17,315 (68%)	<0.001
Diabetes	9,316 (35%)	509 (32%)	8,807 (35%)	0.86
Dyslipidaemia	15,395 (57%)	756 (48%)	14,639 (58%)	<0.001
Current smoking status	10,786 (40%)	604 (39%)	10,182 (40%)	0.53
Chronic kidney disease	4,304 (16%)	382 (24%)	3,922 (15%)	<0.001
Dialysis	694 (2.6%)	52 (3.3%)	642 (2.5%)	0.019
History of PCI	3,259 (12.1%)	191 (12.2%)	3,068 (12.1%)	0.005
History of coronary artery bypass grafting	376 (1.4%)	31 (2.0%)	345 (1.4%)	<0.001
History of heart failure	1,410 (5.2%)	144 (9.2%)	1,266 (5.0%)	<0.001
History of myocardial infarction	2,483 (9.2%)	167 (10.7%)	2,316 (9.1%)	<0.001
Chronic obstructive pulmonary disease	636 (2.4%)	41 (2.6%)	595 (2.3%)	0.29
Abdominal aortic aneurysm/peripheral artery disease	898 (3.3%)	63 (4.0%)	835 (3.3%)	0.042
Diagnosis				0.028
STEMI	21,217 (79%)	1,270 (81%)	19,947 (79%)	
Non-STEMI	5,692 (21%)	297 (19%)	5,395 (21%)	
Presentation on arrival				
Cardiopulmonary arrest	1567 (5.8%)	1567 (100%)	0 (0%)	<0.001
Cardiogenic shock	2,833 (11%)	1,154 (74%)	1,679 (6.6%)	<0.001
Target coronary artery				
Right coronary artery	10,575 (39%)	553 (35%)	10,022 (40%)	<0.001
LMT–left anterior descending artery	13,923 (52%)	983 (63%)	12,940 (51%)	<0.001
Left circumflex artery	5,031 (19%)	321 (20%)	4,710 (19%)	0.061
Bypass graft	74 (0.3%)	3 (0.2%)	71 (0.3%)	0.52
Multivessel disease	10,596 (39%)	676 (43%)	9,920 (39%)	<0.001
LMT lesion	1,049 (3.9%)	168 (11%)	881 (3.5%)	<0.001
Access site				<0.001
Transfemoral intervention	8,485 (32%)	1,009 (64%)	7,476 (30%)	
Transradial intervention	17,680 (66%)	489 (31%)	17,191 (68%)	
Others	744 (2.8%)	69 (4.0%)	675 (3.3%)	
Devices				
Drug‐eluting stent	23,166 (86%)	1,327 (85%)	21,839 (86%)	0.097
Bare‐metal stent	267 (1.0%)	17 (1.1%)	250 (1.0%)	0.70
Drug‐coated balloon	1,740 (6.5%)	86 (5.5%)	1,654 (6.5%)	0.10
Rotational atherectomy	193 (0.7%)	15 (1.0%)	178 (0.7%)	0.25
Mechanical support devices				
IABP	1,218 (4.5%)	249 (15.9%)	969 (3.8%)	<0.001
ECMO	228 (0.8%)	159 (10.1%)	69 (0.3%)	<0.001
Impella	24 (0.1%)	7 (0.4%)	17 (0.1%)	<0.001

CA = cardiac arrest, PCI = percutaneous coronary intervention, STEMI = ST-elevation myocardial infarction, LMT = left main trunk, IABP = intra-aortic balloon pump, ECMO = extracorporeal membrane oxygenation

### Clinical outcomes

**Fig. 2:**
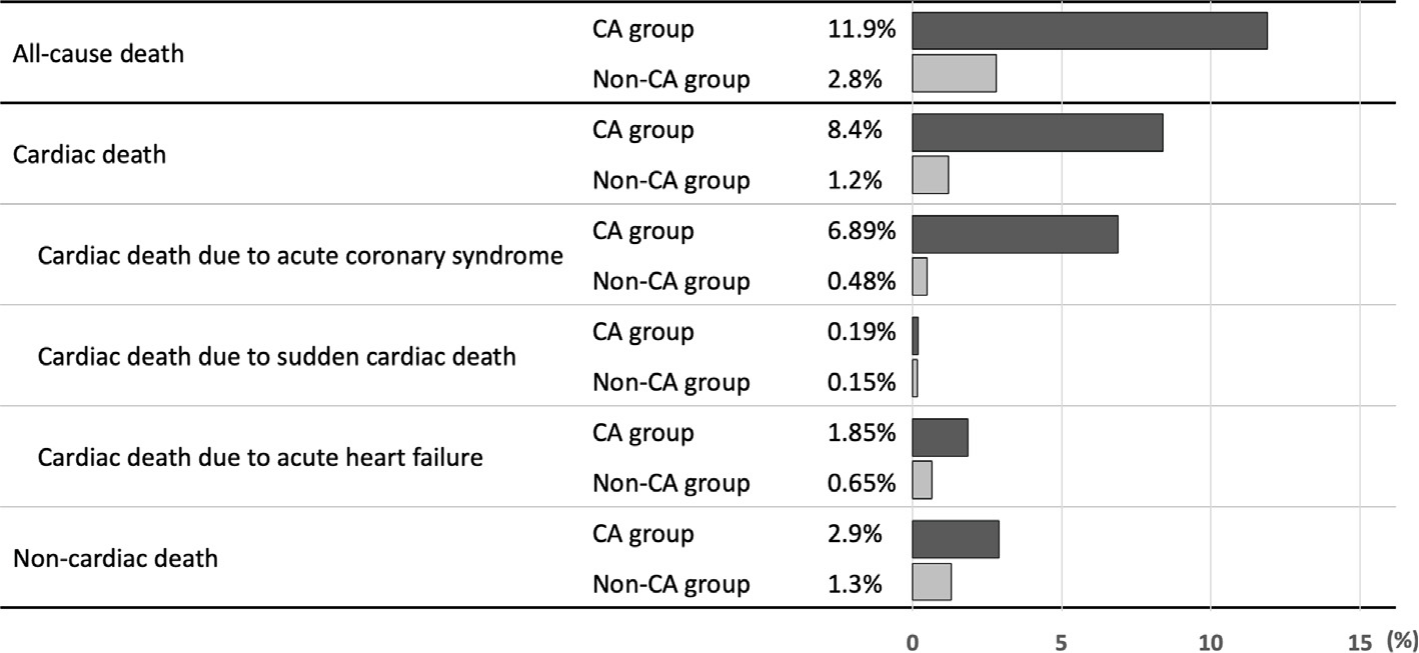
**Cause of death in patients with AMI who survived for 30 days.** Patients with CA had a 7-fold higher risk of cardiac death and a 2-fold higher risk of non-cardiac death than patients without CA. Among the patients with cardiac death, acute coronary syndrome was the predominant cause of death in patients with CA. AMI, acute myocardial infarction; CA, cardiac arrest.

**Fig. 3:**
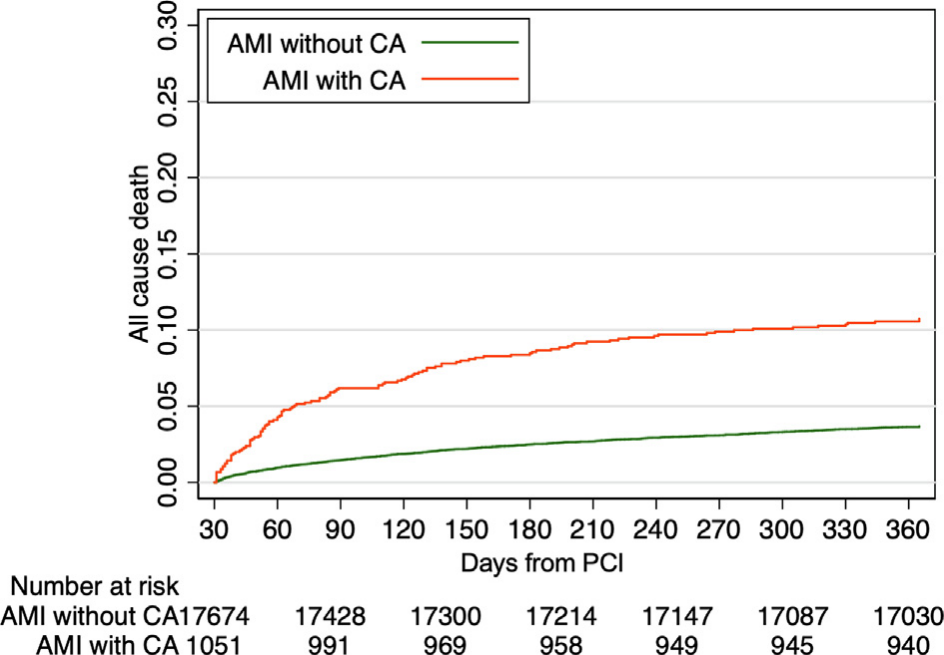
**Time-to-event curve for all-cause death.** All cause death occurred in 186 patients in the CA group and 713 patients in the non-CA group (11.9% vs. 2.8%, *p* < 0.001). AMI, acute myocardial infarction; CKD, chronic kidney disease; PCI, percutaneous coronary intervention.

Clinical outcomes from 30 days to 1 year after PCI are summarized in Table 2 and Fig. 2, and the Kaplan–Meier curve for all-cause death is shown in Fig. 3. All-cause death occurred in 186 patients in the CA group and in 713 patients in the non-CA group (11.9% vs. 2.8%, *p* < 0.001). The all-cause mortality curves for the two groups diverged during the first 6 months, remaining nearly parallel thereafter. In patients with CA, cardiac death was the leading cause of death, with a significantly higher incidence than that in patients without CA (8.4% vs. 1.2%, *p* < 0.001). However, the incidence of non-cardiac death was also significantly higher in patients with CA than in those without CA (2.9% vs. 1.3%, *p* < 0.001). The results of subgroup analyses according to age are shown in Supplementary Figure. In all three age groups, all-cause mortality was consistently higher in patients with CA than in those without CA. The results of univariable and multivariable analyses are presented in Table 3. CA was an independent predictor of all-cause death from 30 days to 1 year after primary PCI (hazard ratio: 2.94 [95% CI: 2.29–3.76]).

**Table 2:** One-year outcomes.

Characteristics	Total *N* = 26,909	Patients with CA *N* = 1,567	Patients without CA *N* = 25,342	*p* value
All-cause death	899 (3.3%)	186 (11.9%)	713 (2.8%)	<0.001
Cardiac death	430 (1.6%)	132 (8.4%)	298 (1.2%)	<0.001
Cardiac death due to ACS	230 (0.85%)	108 (6.89%)	122 (0.48%)	
Cardiac death due to sudden cardiac arrest	42 (0.16%)	3 (0.19%)	39 (0.15%)	
Cardiac death due to heart failure	193 (0.72%)	29 (1.85%)	164 (0.65%)	<0.001
Cardiac death due to stroke	4 (0.01%)	0 (0.00%)	4 (0.02%)	
Cardiac due to procedural complications	4 (0.01%)	1 (0.06%)	3 (0.01%)	
Cardiac death due to bleeding	8 (0.03%)	2 (0.13%)	6 (0.02%)	
Non-cardiac death	366 (1.4%)	46 (2.9%)	320 (1.3%)	<0.001
Death from an unknown cause	103 (0.4%)	10 (0.6%)	93 (0.4%)	0.092
Definite or probable stent thrombosis	19 (0.07%)	6 (0.38%)	13 (0.05%)	0.18
Non-fatal ACS	336 (1.2%)	18 (1.1%)	318 (1.3%)	0.71
STEMI	146 (0.54%)	11 (0.70%)	135 (0.54%)	
NSTEMI	88 (0.33%)	5 (0.32%)	83 (0.33)	0.064
Unstable angina	111 (0.41%)	3 (0.19%)	108 (0.43%)	
Non-fatal ischemic stroke	116 (0.4%)	4 (0.3%)	112 (0.4%)	0.27
Non-fatal bleeding	415 (1.5%)	31 (2.0%)	384 (1.5%)	0.15
Non-fatal heart failure	560 (2.1%)	38 (2.4%)	522 (2.1%)	0.33
Planned revascularization	4,167 (15.5%)	164 (10.5%)	4,003 (15.8%)	<0.001
Staged PCI	2,406 (8.9%)	104 (6.6%)	2,302 (9.1%)	
PCI for chest discomfort symptoms	480 (1.8%)	21 (1.3%)	459 (1.8%)	
PCI for proven myocardial ischemia	989 (3.7%)	31 (2.0%)	958 (3.8%)	0.16
PCI without symptoms/ischemia	283 (1.1%)	9 (0.6%)	274 (1.1%)	
Coronary artery bypass graft	233 (0.9%)	15 (1.0%)	218 (0.9%)	

CA = cardiac arrest, ACS = acute coronary syndrome, STEMI = ST-elevation myocardial infarction, NSTEMI = non-ST-elevation myocardial infarction, PCI = percutaneous coronary intervention

**Table 3:** Hazard ratios for all-cause death.

Characteristics	Univariable analysis				Multivariable analysis			
	Hazard ratio	Lower 95% CI	Upper 95% CI	*p* value	Hazard ratio	Lower 95% CI	Upper 95% CI	*p* value
CA	3.04	2.48	3.72	<0.001	2.94	2.29	3.76	<0.001
Age	1.06	1.05	1.07	<0.001	1.05	1.04	1.06	<0.001
Male sex	0.71	0.61	0.83	<0.001	1.10	0.91	1.34	0.34
STEMI	0.94	0.79	1.12	0.48				
LMT lesion	2.93	2.32	3.70	<0.001	1.64	1.24	2.17	<0.001
MVD	1.49	1.29	1.72	<0.001	1.13	0.95	1.33	0.17
Hypertension	1.19	1.01	1.42	0.04	0.79	0.65	0.95	0.01
Diabetes	1.43	1.24	1.66	<0.001	1.32	1.11	1.56	<0.001
Dyslipidaemia	0.67	0.58	0.77	<0.001	0.78	0.66	0.92	0.003
Current smoking status	0.61	0.52	0.71	<0.001	1.00	0.83	1.22	0.98
CKD stage 1	Reference				Reference			
CKD stage 2	1.47	1.17	1.84	0.001	1.19	0.93	1.50	0.161
CKD stage 3	4.19	3.37	5.22	<0.001	2.37	1.87	3.01	<0.001
CKD stage 4	6.54	4.62	9.25	<0.001	3.53	2.45	5.08	<0.001
CKD stage 5	8.58	6.49	11.36	<0.001	6.70	5.00	8.98	<0.001

CA = cardiopulmonary arrest, STEMI = ST-elevation myocardial infarction, LMT = left main trunk, MVD = multivessel disease, CKD = chronic kidney disease.

## Discussion

### Primary study findings

The main results were as follows. First, among patients who survived for 30 days after AMI, all-cause mortality at 1 year was higher in patients with CA than in patients without CA. Second, when analysed by the age group, the higher mortality observed in patients with CA was consistent across all age groups. Finally, CA was an independent predictor of all-cause mortality after adjustments for other confounders. To the best of our knowledge, the present study includes the most recent and largest-scale analysis evaluating post-discharge outcomes in patients with CA.

### Baseline demographics in patients with CA

Patients with CA were younger and more likely to be male and had a lower prevalence of traditional coronary risk factors (e.g., hypertension and diabetes) but a higher prevalence of CKD and dialysis than patients without CA. It may seem counterintuitive that patients with CA are younger. Because our registry data included only patients who underwent PCI, it cannot be excluded that older CA patients avoided PCI and younger CA patients underwent aggressive PCI, which may have influenced these results. However, previous studies have consistently reported that younger patients are at higher risk for CA. In a report using the J-PCI registry, younger patients had a lower prevalence of traditional coronary risk factors such as hypertension, diabetes, and CKD, but had a higher incidence of CA complications at the time of AMI.

### Impact of concomitant CA on mortality

In previous studies, post-discharge mortality in patients with and without CA is comparable.^16^, ^17^ However, the present study showed that the 1-year mortality was significantly higher in patients with CA than in patients without CA. Several differences between the present study and previous studies should be addressed. First, the patient cohorts may have been enrolled at different time periods. Patients in the present study were enrolled recently, and differences may reflect the fact that modern PCI procedures and *peri*-resuscitation care were provided to the patients. In recent years, there has been a growing emphasis on prehospital cardiopulmonary resuscitation, appropriate application of therapeutic hypothermia, and improvements in mechanical circulatory support devices. These developments have contributed to an increase in the rate of successful discharge for critically ill patients who have experienced CA.^26^, ^27^, ^28^ However, it is important to note that some of these patients may be discharged with a compromised neurological status, which can subsequently lead to adverse outcomes, including death. Previous studies have demonstrated a strong association between significant neurological disability at the time of discharge following CA and poor long-term prognosis.^29^ Second, the number of patients enrolled in the present study differed from that in previous studies.^16^, ^17^ This study enrolled a large number of patients, and data were collected on a nationwide basis, minimizing the possibility of selection bias. The management of critically ill patients differs among facilities, resulting in marked differences in outcomes. Additionally, the generalizability of results is difficult for studies involving only a limited number of patients from a limited number of centres.

### Cause of death after discharge

A unique feature of the present study is that it also includes information on the cause of death. Patients with CA had a 7-fold higher risk of cardiac death and a 2-fold higher risk of non-cardiac death than patients without CA. ACS was found to be the predominant cause of cardiac death in patients with CA. The high incidence of ACS in this population can be attributed to various factors. Stent thrombosis is a phenotype of ACS, and the presence of CA may have increased the risk of developing stent thrombosis. In retrospective studies, the incidence of stent thrombosis in patients with CA undergoing PCI ranged from 2.7% to 31.2%.^30^, ^31^, ^32^ Moreover, although controversial, therapeutic hypothermia may increase the risk of stent thrombosis^30^, ^31^, ^32^ and microcirculatory and endothelial dysfunction after CA.^33^, ^34^, ^35^ Another concerning issue is the low rate of PCI performed after CA. Recent randomized trial findings indicated the benefits of achieving complete revascularization with PCI for non-culprit lesions in patients with STEMI,^36^, ^37^, ^38^, ^39^ and observational studies showed positive outcomes associated with complete revascularization in patients with CA.^40^, ^41^ However, there appears to be therapeutic nihilism and a lack of understanding regarding early neurological prognostication after CA, which may contribute to underutilization of PCI in this population. The higher incidence of non-cardiac death in patients with CA should also be noted. Patients with CA usually have a high burden of pre-arrest comorbidities, which may contribute to a poor prognosis after discharge.^42^, ^43^ In addition, the severity of neurological dysfunction caused by CA is associated with an increased incidence of mortality after discharge.^29^ The high prevalence of comorbidity in patients with CA might be associated with high mortality. In the present study, patients with CA had a higher prevalence of CKD and dialysis than patients without CA. Complex lesions such as multivessel disease and left main lesions are also more common in patients with CA. All of these factors have been reported to be associated with worse clinical outcomes in patients with AMI. In addition to the direct effect of CA, the impact of these comorbidities on mortality should also be considered.

### Clinical implications

Previous investigations have focused on acute-phase events (in-hospital mortality) after initial treatment of CA. However, assessments of the long-term prognosis of patients with CA are essential to facilitate decision-making and patient education. The present study found that patients with CA were the most vulnerable within the first 6 months after discharge, when the majority of all-cause deaths occurred. Considering that ACS is a major cause of mortality in patients with CA, there may be potential benefits in ensuring appropriate management. This includes the administration of optimal medical therapy and, when necessary, consideration of revascularization for any residual lesions. Timely and appropriate interventions may play a role in improving outcomes in this population. In addition, comorbidities present prior to the onset of CA may also be associated with high mortality after CA, so aggressive management of these comorbidities may also be important.

### Limitations

The results of this study should be interpreted considering some limitations. First, this registry does not include information on in-hospital mortality within 30 days. This is because the registry includes information only on patients who are discharged alive. Therefore, we could not evaluate clinical events that occurred in the acute phase. Second, patients with CA are likely to have various complications. Among these, post-CA brain injury is the major cause of death and long-term disability in patients resuscitated following CA. However, the registry does not have data on the presence or absence of brain injury or its severity; therefore, it lacks important information that may explain the higher mortality rate in patients with CA. Third, although optimal medical therapy is essential for patients with AMI, this registry lacks information on prescribed medications. Therefore, we were unable to compare the achievement of optimal medical therapy between patients with and without CA. Fourth, information regarding specific treatments for patients presenting with CA, such as therapeutic hypothermia or implantable cardioverter defibrillators, is unavailable. There is also no information on the location of the onset of CA. This information could be crucial when considering the prognosis of patients with CA. Fifth, we recognize that measures of comorbidity severity, such as the Charlson comorbidity index, could provide additional insight into the impact and severity of comorbid conditions on patient outcomes. However, due to the design of the registry, the specific data needed to calculate these indices were not collected. This limitation precludes a comprehensive analysis of the impact of morbidity on patient mortality, which should be considered when interpreting our results. Finally, prognostic data were collected by staff at each site. The completeness and accuracy of data collection was not monitored, so the possibility of missing data or errors cannot be excluded.

## Conclusion

Compared with patients without CA, patients with concomitant CA at the onset of AMI have a worse prognosis after hospital discharge.

## CRediT authorship contribution statement

**Hirohiko Ando:** Writing – original draft, Methodology, Conceptualization. **Mitsuaki Sawano:** Writing – review & editing, Formal analysis, Data curation. **Shun Kohsaka:** Writing – review & editing, Supervision, Conceptualization. **Hideki Ishii:** Writing – review & editing, Supervision. **Atomu Tajima:** Writing – review & editing, Validation. **Wataru Suzuki:** Writing – review & editing, Validation. **Ayako Kunimura:** Writing – review & editing, Validation. **Yusuke Nakano:** Writing – review & editing, Validation. **Ken Kozuma:** Writing – review & editing, Supervision. **Tetsuya Amano:** Writing – review & editing, Supervision.

## Declaration of competing interest

The authors declare the following financial interests/personal relationships which may be considered as potential competing interests: ‘Dr. Kohsaka has received speaker fees from Bristol-Myers Squibb and Pfizer and has received institutional research grant support from Novartis and AstraZeneca. Dr. Ishii received lecture fees from Astellas Pharma, AstraZeneca, Bayer Pharmaceutical Co., Ltd., Chugai Pharma, Inc., Daiichi Sankyo, and MSD K. K. Dr. Amano received lecture fees from Astellas Pharma, AstraZeneca, Bayer, Daiichi Sankyo, and Bristol-Myers Squibb. The remaining authors have no conflicts of interest to declare’.

## Appendix A Supplementary material

Supplementary Figure **Time-to-event curves for all-cause death by age group.** In all three age groups, all-cause mortality was consistently higher in patients with CA than in those without CA. AMI, acute myocardial infarction; CA, cardiac arrest; PCI, percutaneous coronary intervention.
