# Supplementary figures and images for: Cardiac arrest and post-discharge mortality in patients with myocardial infarction: A large-scale nationwide registry analysis
Source: Resusc Plus. 2024 May 3;18:100647. doi: 10.1016/j.resplu.2024.100647 (PMC11088348; doi:10.1016/j.resplu.2024.100647)

### <60 years

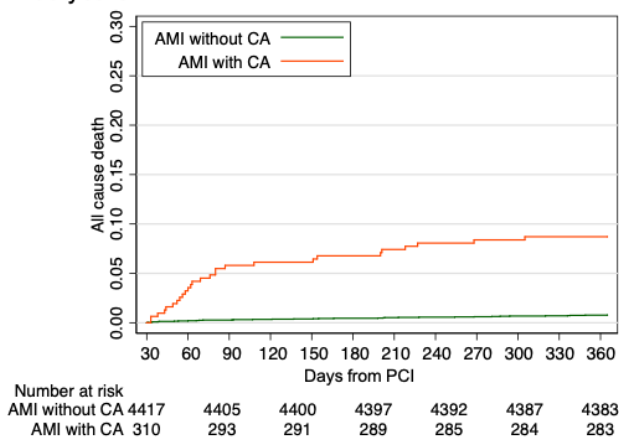

### 61-70 years

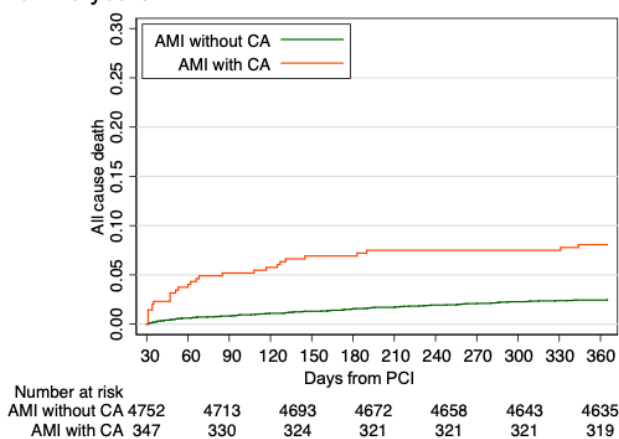

### 71-80 years

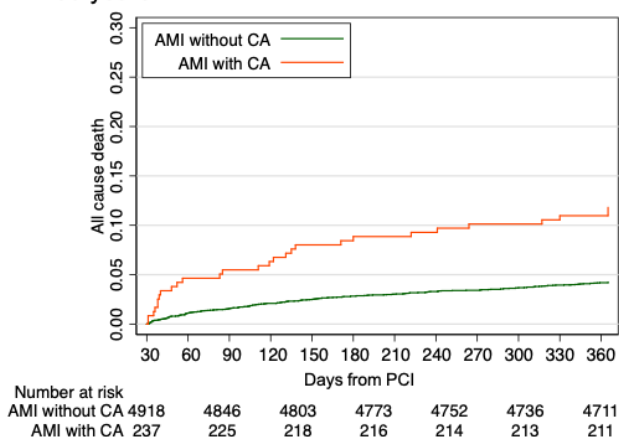

Supplement: Supplementary Figure — Time-to-event curves for all-cause death by age group. In all three age groups, all-cause mortality was consistently higher in patients with CA than in those without CA. AMI, acute myocardial infarction; CA, cardiac arrest; PCI, percutaneous coronary intervention. [file mmc1.pdf]
